# *Porphyromonas gingivalis* diffusible signaling molecules enhance *Fusobacterium nucleatum* biofilm formation *via* gene expression modulation

**DOI:** 10.1080/20002297.2023.2165001

**Published:** 2023-01-10

**Authors:** Yukiko Yamaguchi-Kuroda, Yuichiro Kikuchi, Eitoyo Kokubu, Kazuyuki Ishihara

**Affiliations:** a Department of Endodontics, Tokyo Dental College, 2-9-18 Kanda-Misakicho, Tokyo 101-0061, Chiyoda-ku, Japan; b Department of Microbiology, Tokyo Dental College, 2-1-14 Kanda-Misakicho, Tokyo 101-0061, Chiyoda-ku, Japan

**Keywords:** Synergy, biofilm, periodontal disease, Fap2, *Porphyromonas gingivalis*, *Fusobacterium nucleatum*

## Abstract

**Background:**

Periodontitis is caused by a dysbiotic shift in the dental plaque microbiome. *Fusobacterium nucleatum* is involved in the colonization of *Porphyromonas gingivalis*, which plays a key role in dysbiosis, via coaggregation and synergy with this microorganism.

**Aim:**

We investigated the effect of diffusible signaling molecules from *P. gingivalis* ATCC 33277 on *F. nucleatum* TDC 100 to elucidate the synergistic mechanisms involved in dysbiosis.

**Methods:**

The two species were cocultured separated with an 0.4-µm membrane in tryptic soy broth, and *F. nucleatum* gene expression profiles in coculture with *P. gingivalis* were compared with those in monoculture.

**Results:**

RNA sequencing revealed 139 genes differentially expressed between the coculture and monoculture. The expression of 52 genes was upregulated, including the coaggregation ligand-coding gene. Eighty-seven genes were downregulated. Gene Ontology analysis indicated enrichment for the glycogen synthesis pathway and a decrease in de novo synthesis of purine and pyrimidine.

**Conclusion:**

These results indicate that diffusible signaling molecules from *P. gingivalis* induce metabolic changes in *F. nucleatum*, including an increase in polysaccharide synthesis and reduction in de novo synthesis of purine and pyrimidine. The metabolic changes may accelerate biofilm formation by *F. nucleatum* with *P. gingivalis*. Further, the alterations may represent potential therapeutic targets for preventing dysbiosis.

## Introduction

Periodontitis is a highly prevalent disease, with a global age-standardized prevalence of approximately 10% [1]. Inflammation caused by this disease disrupts the periodontal ligament, induces resorption of the alveolar bone, and results in tooth loss [2,3]. A major feature of this disease is dysbiosis, which is a shift in the composition and abundance of the subgingival microbiota toward more pathogenic species [4]; however, the details of the dysbiotic shift are yet to be elucidated. Understanding the intricacies of dysbiosis is essential for developing treatment and prevention strategies for this condition. Periodontopathic bacteria implicated in dysbiosis include *Porphyromonas gingivalis*, *Tannerella forsythia*, *Treponema denticola*, and *Filifactor alocis*, which are abundant in periodontitis lesions [5–7]. Among them, *P. gingivalis* is a key microorganism for the induction of this dysbiotic shift of the microbiome [8].

Coaggregation, which is adherence between bacteria, is essential for dental plaque formation [9–11]. *Fusobacterium nucleatum* is one of the most frequently detected bacterial species in both the healthy oral microbiome and periodontal disease lesions. This microorganism is associated with systemic conditions such as preterm birth and colorectal cancer [5,12–14]. In an *in vivo* study, *F. nucleatum* was detected between the basal layer and top layer of the dental plaque, and *P. gingivalis* was detected in the top layer [15]. In another *in vivo* study, *Fusobacterium* was detected in the annulus area between the tooth side and peripheral area of the plaque. *Porphyromonas* was detected in the peripheral area and was found adjacent to *Fusobacterium* [16]. *In vitro* studies revealed that *F. nucleatum* may coaggregate with periodontopathic bacteria such as *P. gingivalis*, *T. denticola*, and *Prevotella intermedia* [17–19] and exert a synergistic effect with periodontopathic bacteria, including *P. gingivalis*, on pathogenicity or biofilm formation [18,20–22]. *F. nucleatum* has a coaggregation ligand Fap2 that mediates the adhesion of *F. nucleatum* to *P. gingivalis* and red blood cells [17] and promotes the growth of *P. gingivalis* in aerobic and CO_2_ depleted conditions [23]. The localization of *F. nucleatum* and *P. gingivalis* in the plaque structure and their coaggregation and synergy indicate that *F. nucleatum* assists the colonization of *P. gingivalis*. In addition, Feuille et al. found an increase in the virulence of *P. gingivalis* cocultured with *F. nucleatum* [24]. Metzger et al. also reported the synergistic pathogenicity of *P. gingivalis* and *F. nucleatum* using a murine subcutaneous chamber model [21]. Recent proteomic analyses have provided insights into synergic interactions *via* cross-feeding among species such as *Streptococcus gordonii*, *F. nucleatum*, and *P. gingivalis* using a chemically defined medium or brain heart infusion broth [25,26]. In these studies, *S. gordonii* showed synergism with *F. nucleatum* by providing ArcD-regulated ornithine as an energy source, whereas *F. nucleatum* facilitated *P. gingivalis* biofilm formation by providing putrescine. These studies provide insights into how dysbiosis is involved in biofilm formation. However, the effect of *P. gingivalis* on *F. nucleatum* was not fully investigated.

Previously, we showed that *F. nucleatum* TDC 100 isolated from apical periodontitis lesions strongly adheres to type-I collagen and shows enhanced biofilm formation through a synergistic relationship with *P. gingivalis* [27]. Coinfection of these species also enhanced the invasion of epithelial cells by *P. gingivalis* [28,29]. Enhanced *F. nucleatum* biofilm formation by *P. gingivalis* was observed in a coculture separated with an 0.4-µm membrane, which was significantly higher than that observed for other bacterial species [27], indicating that a factor produced by *P. gingivalis* plays an important role in the synergy between *F. nucleatum* and *P. gingivalis*. Therefore, in this study, we investigated the effect of diffusible signaling molecules of *P. gingivalis* on the mRNA and protein expression in *F. nucleatum* to clarify the mechanism of enhanced *F. nucleatum* biofilm formation by *P. gingivalis*.

## Materials and methods

### Bacterial strains and culture conditions

*F. nucleatum* TDC 100 [27] and *P. gingivalis* ATCC 33277 [30] were maintained on tryptic soy agar (BD Bioscience, San Jose, CA) containing 10% defibrinated horse blood, 5 µg/mL hemin, and 0.5 µg/mL menadione. All strains were cultivated at 37°C under anaerobic conditions (H_2_: 10%, CO_2_: 10%, N_2_: 80%) in an anaerobic chamber (ANX-4; Hirasawa, Tokyo, Japan).

### Induction of biofilm formation using a two-compartment system

The interaction of the diffusible signaling molecules between *F. nucleatum* and *P. gingivalis* was evaluated via coculture separated with an 0.4-µm membrane using a two-compartment system as described previously [27]. *F. nucleatum* and *P. gingivalis* were inoculated into tryptic soy broth (TSB) containing 5 µg/mL hemin and 0.5 µg/mL menadione (TSBhm) and precultured anaerobically for 1 day at 37°C. Then, each culture was diluted with fresh TSBhm to an OD 660 = 0.1 and cultured anaerobically. After 24 h, each culture was diluted 1:2 with fresh TSBhm. Next, 750 µL of the resulting *F. nucleatum* culture was added into the wells (lower well) of a 12-well plate, coated with type-I collagen and polystyrene (Iwaki, Tokyo, Japan). Upper wells (Transwell, Corning, NY) were then inserted into the lower well, followed by the addition of 750 µL of the resulting *P. gingivalis* culture to the upper wells. The organisms were then cocultured while being physically separated by an 0.4-µm pore size membrane. For monoculture, only 750 µL of TSBhm was added to the upper well. After anaerobic incubation at 37°C for 2 days, the upper wells were removed. Biofilm formation was evaluated using crystal violet staining with Spectra MAX M5 (Molecular Device, Sunnyvale, CA) [31].

### F. nucleatum biofilm protein profiling

*F. nucleatum* biofilms were lysed with a sample buffer containing a reducing agent, and the lysate proteins were resolved using 10–20% sodium dodecyl sulfate-polyacrylamide electrophoresis (SDS-PAGE). The protein profiles were determined by staining the gel with Coomassie Blue. In the cocultured *F. nucleatum* protein profile, the 39-kDa protein showed considerably enhanced staining intensity. To identify the protein band, the electrophoresed bands were transferred from the gel to a polyvinylidene difluoride (PVDF) membrane (Immobilon-P; Millipore, Billerica, MA) using Trans-Blot SD Semi-Dry Transfer Cell (Bio-Rad, Hercules, CA). After staining the membrane with Coomassie Blue, the 39-kDa protein band was excised from the membrane, and the N-terminal amino acids were sequenced using the automatic peptide sequencer Procise 494 cLC (Applied Biosystems, Foster City, CA). The peptide sequences were matched with the genomic sequences of *F. nucleatum* in the NCBI database (http://blast.ncbi.nlm.nih.gov).

Based on the obtained gene sequence, an antibody against the protein was prepared. The peptide ESEVKNWRWQPTAW showed the highest similarity to residues 352–365 of *Fusobacterium periodonticum* outer membrane protein A (FomA), which has an additional N-terminal Cys residue. This sequence was identical between *F. nucleatum* and *F. periodonticum* except for the sixth residue. The peptide was synthesized using F-moc chemistry and was conjugated to keyhole limpet hemocyanin using maleimidobenzoic acid N-hydroxy succinimide ester, and the antibody was prepared as described previously [32]. To quantify FomA, immunoblotting was performed as described earlier. Ten micrograms of biofilm organized in the lower well, which was monocultured and cocultured with *P. gingivalis*, was separated using SDS-PAGE and transferred onto a PVDF membrane. FomA was stained with anti-FomA antibody (1:1,000 dilution) and goat anti-rabbit IgG (1:3,000 dilution). The membrane was scanned, and the staining intensity of the developed 39-kDa bands was evaluated using Image-Quant TL software v.8.1 (GE Healthcare, Little Chalfont, UK).

### RNA-sequencing

*F. nucleatum* TDC 100 was grown as a monoculture and cocultured with *P. gingivalis* ATCC 33277 for 2 days using the two-compartment system. After removing the upper wells, three wells containing *F. nucleatum* were combined into one sample for RNA extraction, and the total RNA was isolated using TRIzol (ThermoFisher Scientific, Tokyo, Japan). The total RNA was obtained from three independent replicates of monocultured and cocultured *F. nucleatum*. DNA was removed using the TURBO DNA-free kit (Thermo Fisher Scientific, Waltham, MA), and ribosomal RNA was extracted from the sample using the Ribo-Zero rRNA Removal kit (Bacteria) (Illumina). A library was constructed from the extracted RNA using the Truseq Stranded mRNA Sample Prep kit (Illumina) according to the manufacturer’s instructions. The obtained library was sequenced on HiSeq 2500 (Illumina); 100-bp paired-end reads were trimmed using cutadapt (https://cutadapt.readthedocs.org/en/stable/) and Trimmomatic (http://www.usadellab.org/cms/index.php?page=trimmomatic). As the genomic DNA of *F. nucleatum* TDC 100 has not been sequenced, the genomes of *F. nucleatum* subsp. *nucleatum* ATCC 25586, *F. nucleatum* subsp. *vincentii* ATCC 49256, *F. nucleatum* subsp. *vincentii* 3_1_36A2, and *F. nucleatum* KCOM 2931 were used as references to map the obtained sequences. Among these subspecies, *F. nucleatum* KCOM 2931 showed the highest mapping rate (89.7%), and its 16S rRNA sequence showed 99% identity with that of *F. nucleatum* TDC 100. Thereafter, the obtained sequences were mapped using TopHat (http://ccb.jhu.edu/software/tophat/index.shtml) and Bowtie1 (http://bowtie-bio.sourceforge.net/index.shtml) with *F. nucleatum* KCOM 2931 as the reference strain. From the mapped sequences, the gene expression in monocultured and cocultured *F. nucleatum* was evaluated using cufflinks and cuffdiff. The difference in expression between monoculture and coculture was compared using TCC-GUI [33]. Normalization was carried out using the TMM method, and differentially expressed genes were identified using edgeR [34], where findings with a p-value of 0.05 were considered significant and the false discovery rate (FDR) was set to 0.05, with differences in gene expression evaluated using log2 fold ≥1.5. Differentially expressed genes were subjected to Gene Ontology (GO) analysis using goatools v. 1.1.6 [35].

### Statistical analysis

To investigate the effect of coculture on biofilm formation, the level of biofilm formation and FomA expression were analyzed using Student’s *t*-test at a 5% level of significance.

## Results

### Effect of P. gingivalis coculture on F. nucleatum protein profile

**Figure 1.**
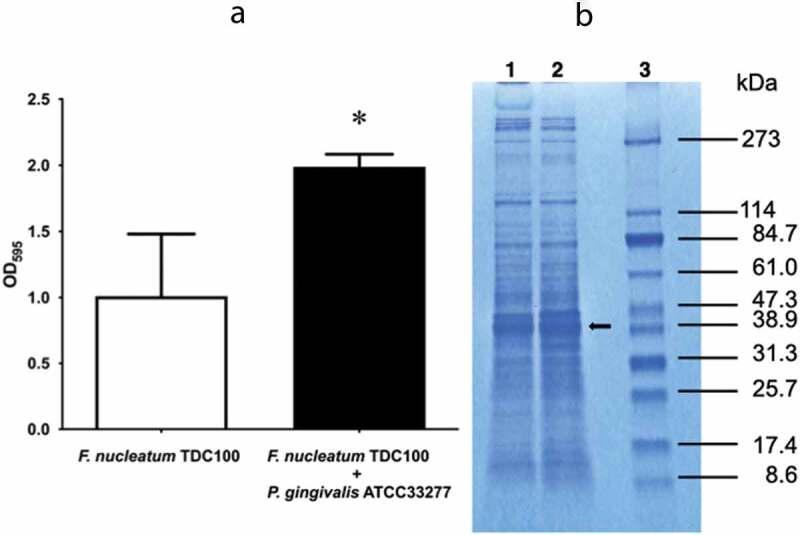
Biofilm formation by *Fusobacterium nucleatum* with/without *Porphyromonas gingivalis* in a two-compartment assay system (a) and the protein profile determined using sodium dodecyl sulfate polyacrylamide gel electrophoresis (b). (a) Biofilm mass of cocultured *F. nucleatum* TDC 100 evaluated using crystal violet staining. Student’s *t*-test was used for inter-group comparisons. *p < 0.05. Data are shown as mean ± standard deviation. (b) Lanes 1: *F. nucleatum* TDC 100 alone; 2: *F. nucleatum* TDC 100 with *P. gingivalis* ATCC 33277; 3: molecular size marker. Arrow indicates the 39-kDa protein.

**Figure 2.**
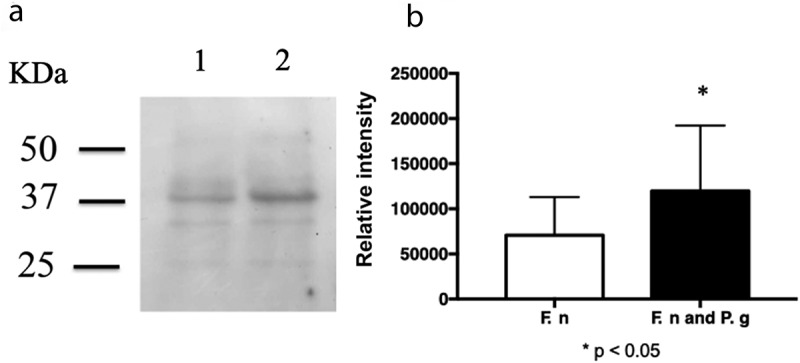
Quantification of FomA using immunoblotting. (a) Immunoblot analysis of *Fusobacterium nucleatum* TDC 100 cocultured with *Porphyromonas gingivalis* ATCC 33277. Lanes 1: *F. nucleatum* TDC 100 alone; 2: *F. nucleatum* TDC 100 with *P. gingivalis* ATCC 33277. The blotted membrane was detected using an antibody against *F. nucleatum* FomA, and the reacted band was detected using horseradish peroxidase conjugated goat anti-rabbit antibody. (b) A comparison of the intensity of the 39-kDa bands. Data are shown as mean ± standard deviation. *p < 0.05 by Student’s *t*-test.

Biofilm formation under *P. gingivalis* coculture was greater than that under *F. nucleatum* TDC 100 monoculture (Figure 1b, *p* < 0.01). A comparison of protein profiles revealed a band at approximately 39 kDa with increased staining intensity for *F. nucleatum* TDC 100 cocultured with *P. gingivalis* ATCC 33277 (Figure 1b). The N-terminal amino acid sequence of the band at 39 kDa was EVTPAWRPNG. The BLAST analysis revealed that this sequence was identical to that of residues 49–58 of FomA. The predicted molecular mass of amino acids 1–48 was 5179.17, indicating that the 39-kDa band was FomA without these 48 amino acid residues. The antibody reactivity to the 39-kDa band (Figure 2a) also supported this result.

The level of the major outer membrane protein FomA was investigated using immunoblotting with rabbit anti-FomA antibody. Image analysis of the immunoblots using Imagequant TL showed that staining of the 39-kDa and faint 35-kDa bands detected with the anti-FomA antibody in cocultured *F. nucleatum* was 1.7 times more intense than that for *F. nucleatum* monoculture (Figure 2b).

### RNA-sequencing of F. nucleatum TDC 100 cocultured with P. gingivalis ATCC 33277

**Figure 3.**
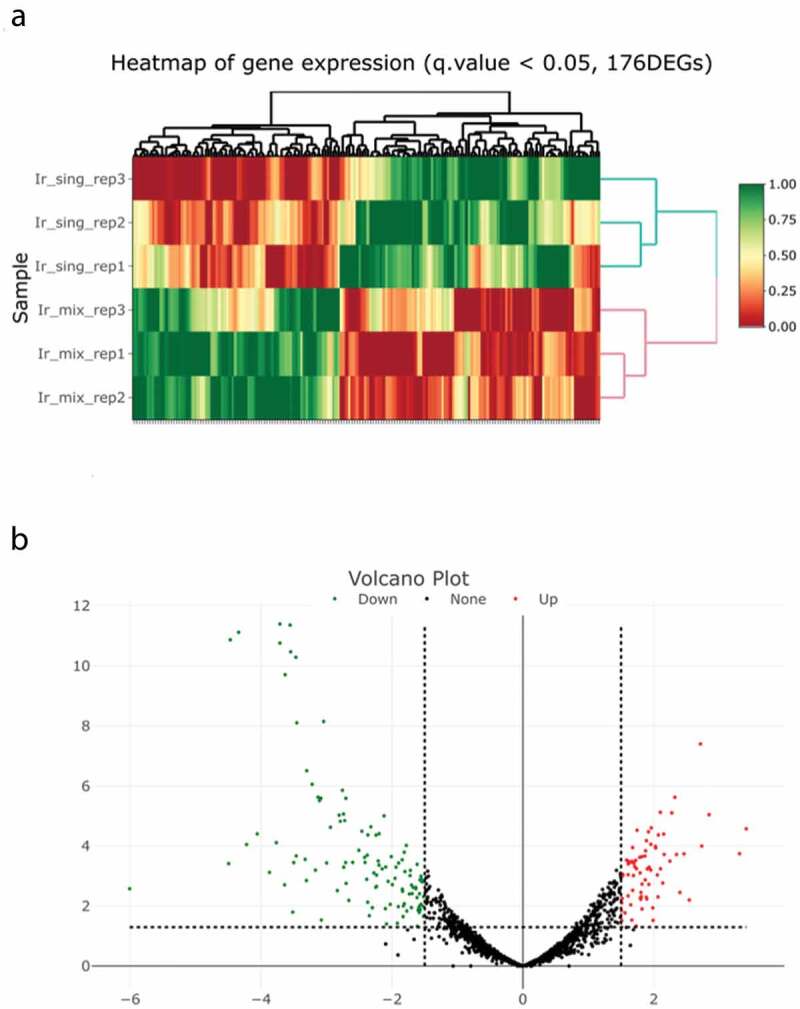
Genes differentially expressed between cocultured and monocultured *Fusobacterium nucleatum*. (a) Hierarchical clustering of differentially expressed genes at a false discovery rate (FDR) of <0.05 between cocultured and monocultured *F. nucleatum*. Ir_sing: monocultured, Ir_mix: cocultured, red to green color scale indicates the relative level of gene expression. (b) Volcano plots show differentially expressed genes (*p* < 0.05, FDR <0.05). Horizontal dotted lines show the p-value cut-off of 0.05, and vertical dotted lines show the expression level log2 (fold change) cut-off of 1.5. Red and green dots indicate upregulated and downregulated, respectively.

To evaluate the effect of *P. gingivalis* on *F. nucleatum* in coculture, RNA-sequencing analysis was performed. At FDR <0.05, 176 differentially expressed genes were obtained (Figure 3a). Among these, the genes from the coculture showing a log2-fold difference of >1.5 compared with their expression in the monoculture of *F. nucleatum*, *p* < 0.05, and FDR <0.05 were selected (Figure 3b). In *F. nucleatum* cocultured with *P. gingivalis*, 139 genes (4.9%) were differentially expressed compared with those in monocultured *F. nucleatum*. Among them, 52 genes were upregulated (Table 1), including the gene encoding the galactose inhibitable autotransporter adhesion Fap2, a protein involved in porphyrin metabolism (*cobK* and Cobart-precorrin 5A hydrolase), chaperone proteins (ClpB, DnaK, and DnaJ), proteins involved in polysaccharide synthesis (glycogen synthase, GlgG, glucose-1-phosphate adenylyltransferase, 1,4-alpha-glucan branching protein GlgB, and GalU), and proteins involved in membrane transport (basic amino acid ABC transporter substrate-binding protein, amino acid ABC transporter ATP-binding protein, DMT family transporter, AzlC family ABC transporter permease, branched-chain amino acid transporter permease, basic amino acid ABC transporter substrate-binding protein, efflux RND transporter permease subunit, ABC transporter ATP-binding protein, energy-coupling factor transporter transmembrane protein EcfT, autotransporter serine protease fusolisin, ABC transporter permease, and ABC transporter ATP-binding protein). Interestingly, the expression of *fomA* did not show an increase in the RNA-sequencing.

**Table 1.** Upregulated genes in cocultured *Fusobacterium nucleatum.*

Gene no. or ID	Annotation	log_2_(co/mono*)
CS401_RS09410	Hypothetical protein	3.414
*clpB*	ATP-dependent chaperone ClpB	3.312
CS401_RS03075	Basic amino acid ABC transporter substrate-binding protein	2.843
DnaK	Molecular chaperone DnaK	2.733
CS401_RS01585	Glucose-1-phosphate adenylyltransferase	2.713
CS401_RS03070	M20 family metallopeptidase	2.462
CS401_RS09415	Hypothetical protein	2.399
*metK*	Methionine adenosyltransferase	2.346
CS401_RS01590	1,4-alpha-glucan branching protein GlgB	2.323
CS401_RS05055	Galactose inhibitable autotransporter adhesin Fap2	2.276
CS401_RS06090	V-type ATP synthase subunit D	2.246
CS401_RS06310	FAD-dependent oxidoreductase	2.159
CS401_RS07400	Energy-coupling factor transporter transmembrane protein EcfT	2.151
CS401_RS06095	V-type ATP synthase subunit B	2.136
CS401_RS07100	DUF1353 domain-containing protein	2.103
CS401_RS08440	DUF523 domain-containing protein	2.101
CS401_RS03615	Hypothetical protein	2.07
*dnaJ*	Molecular chaperone DnaJ	2.054
CS401_RS07395	Glutathione peroxidase	2.029
CS401_RS00690	MATE family efflux transporter	2.026
CS401_RS9850	Multispecies: ABC transporter ATP-binding protein	1.963
CS401_RS03620	Cytidylate kinase-like family protein	1.956
CS401_RS3610	Efflux RND transporter permease subunit	1.953
CS401_RS07425	GDYXXLXY domain-containing protein	1.93
*raiA*	Ribosome-associated translation inhibitor RaiA	1.925
*glgG*	Glucose-1-phosphate adenylyltransferase subunit GlgD	1.919
CS401_RS07390	ABC transporter ATP-binding protein	1.9
CS401_RS09845	ABC transporter permease	1.887
CS401_RS02470	S1 RNA-binding domain-containing protein	1.876
CS401_RS02460	Branched-chain amino acid transporter permease	1.87
CS401_RS01575	Glycogen synthase	1.865
CS401_RS01335	Basic amino acid ABC transporter substrate-binding protein	1.821
CS401_RS01340	Amino acid ABC transporter ATP-binding protein	1.818
CS401_RS09420	Hypothetical protein	1.804
CS401_RS02465	GNAT family N-acetyltransferase	1.791
CS401_RS2450	DMT family transporter	1.785
CS401_RS08435	AAA family ATPase	1.745
CS401_RS03260	ABC transporter ATP-binding protein/permease	1.736
*cobK*	Precorrin-6A reductase	1.723
CS401_RS07810	Autotransporter serine protease fusolisin	1.71
*cbiG*	Cobalt-precorrin 5A hydrolase	1.695
CS401_RS02455	AzlC family ABC transporter permease	1.689
CS401_RS03215	AEC family transporter	1.678
CS401_RS03565	Hypothetical protein	1.677
*uvrC*	Excinuclease ABC subunit	1.665
*galU*	UTP-glucose-1-phosphate uridylyltransferase	1.656
CS401_RS08075	Txe/YoeB family addiction module toxin	1.611
*nth*	Endonuclease III	1.611
CS401_RS09665	DNA polymerase III subunit alpha	1.608
CS401_RS02055	Putative DNA modification/repair radical SAM protein	1.585
*metG*	Methionine – tRNA ligase	1.531
CS401_RS08695	CoA transferase subunit A	1.529

*co/mono: coculture/monoculture.

Eighty-seven genes were downregulated (Table 2), including those encoding protein involved in *de novo* synthesis of purine (phosphoribosyl amine-glucine ligase, *purH*, class I SAM-dependent methyltransferase, phosphoribosyl glycinamide formyl transferase, *purM*, amidophosphoribosyltransferase, phosphoribosylaminoimidazole-succinocarboxamide synthase, *purE*, and phosphoribosylformylglycinamidine synthetase), proteins involved in *de novo* pyrimidine synthesis (bifunctional pyr operon transcriptional regulator/uracil phosphoribosyltransferase PyrR, aspartate carbamoyltransferase, dihydroorotase, glutamine-hydrolyzing carbamoyl-phosphate synthase small subunit, carbamoyl-phosphate synthase large subunit, dihydroorotate dehydrogenase electron transfer subunit, dihydroorotate dehydrogenase, orotidine 5’-phosphate decarboxylase, and orotate phosphoribosyltransferase), *bioA* involved in biotin metabolism, and TonB-dependent receptor.

**Table 2.** Downregulated genes in cocultured *Fusobacterium nucleatum.*

Gene no. or ID	Annotation	log_2_(co/mono*)
CS401_RS04350	DUF2194 domain-containing protein	−6.008
*purE*	5-(carboxyamino)imidazole ribonucleotide mutase	−4.496
CS401_RS07440	Queuosine precursor transporter	−4.47
CS401_RS07445	Radical SAM protein	−4.342
CS401_RS04345	Hypothetical protein	−4.221
CS401_RS08470	CoA-disulfide reductase	−4.058
CS401_RS08465	Helix-turn-helix transcriptional regulator	−3.871
CS401_RS07475	Sodium-dependent transporter	−3.767
*purF*	Amidophosphoribosyltransferase	−3.713
CS401_RS02225	Phosphoribosylaminoimidazole-succinocarboxamide synthase	−3.712
CS401_RS04965	Hypothetical protein	−3.641
*bioA*	Adenosylmethionine—8-amino-7-oxononanoate transaminase	−3.633
*purM*	Phosphoribosylfomylglycinamidine cyclo-ligase	−3.557
CS401_RS02215	Hypothetical protein	−3.548
*pelF*	GT4 family glucosyltransferase PelF	−3.501
CS401_RS02235	Phosphoribosylformylglycinamidine synthetase	−3.469
CS401_RS04330	Hypothelical protein	−3.463
CS401_RS02205	Phosphoribosylglycinamide formyltransferase	−3.454
*pelG*	Exopolysaccharide Pel transporter PelG	−3.327
CS401_RS04355	Hypothetical protein	−3.307
CS401_RS10000	Aspartate carbamoyltransferase catalytic subunit	−3.302
*megL*	Methionine gamma-lyase	−3.219
CS401_RS06670	A24 family peptidase	−3.17
CS401_RS10010	Dihydroorotase	−3.13
CS401_RS09550	Na+/H+ antiporter NhaC family protein	−3.086
CS401_RS02200	Class I SAM-dependent methyltransferase	−3.044
CS401_RS04325	Endo alpha-1,4 polygalactosaminidase	−2.998
CS401_RS10045	Hypothetical protein	−2.939
CS401_RS07480	Tyrosine phenol-lyase	−2.834
CS401_RS10015	Glutamine-hydrolyzing carbamoyl-phosphate synthase small subunit	−2.806
*carA*	Orotidine-5’-phosphate decarboxylase	−2.788
*purH*	Bifunctional phosphoribosylaminoimidazolecarboxamide formyltransferase/IMP cyclohydrolase	−2.757
CS401_RS08865	ABC transporter ATP-binding protein/permease	−2.741
CS401_RS05375	6.7-dimethyl-8-ribityllumazine synthase	−2.736
CS401_RS02575	Alpha/beta hydrolase	−2.722
CS401_RS08540	Hypothetical protein	−2.708
CS401_RS00210	TonB-dependent receptor	−2.704
*ribD*	Bifunctional diaminohydroxyphosphoribosylaminopyrimidine deaminase/5-amino-6-(5-phosphoribosylamino)uracil reductase	−2.6
CS401_RS10025	Dihydroorotate dehydrogenase electron transfer subunit	−2.506
CS401_RS10020	Carbamoyl-phosphate synthase large subunit	−2.46
CS401_RS08600	3-aminobutyl-CoA ammonia lyase	−2.419
CS401_RS09220	Transcription antiterminator	−2.411
CS401_RS10030	Dihydroorotate dehydrogenase	−2.377
CS401_RS09160	Cytochrome c biogenesis protein CcdA	−2.368
CS401_RS02190	Phosphoribosylamine – glucine ligase	−2.322
*pflA*	Pyrvate formate-lyase-activating protein	−2.276
CS401_RS05390	Riboflavin synthase	−2.249
*cbpF*	CEACAM-binding trimeric autotransporter adhesin CbpF	−2.244
*pflB*	Formate C-acetyltransferase	−2.235
CS401_RS06640	Hypothelical	−2.234
*nifJ*	Pyruvate:ferredoxin (flavodoxin) oxidoreductase	−2.21
CS401_RS09995	Bifunctional pyr operon transcriptional regulator/Uracil phosphoribosyltransferase PyrR	−2.198
CS401_RS02270	M42 family metallopeptidase	−2.196
*pyrE*	Orotate phosphoribosyltransferase	−2.15
CS401_RS07595	HPr family phosphocarrier protein	−2.121
CS401_RS09080	RNA-binding protein	−2.095
CS401_RS02570	Hypothetical protein	−2.058
CS401_RS08860	ABC transporter ATP-binding protein/permease	−2.011
CS401_RS05160	Hypothetical protein	−1.994
CS401_RS07575	Hypothetical protein	−1.954
CS401_RS08855	Flavodoxin	−1.938
CS401_RS06490	Flavodoxin	−1.898
CS401_RS03225	Acetyl-CoA hydrolase/transferase family protein	−1.844
CS401_RS08625	Hypothetical protein	−1.841
CS401_RS06480	NrdH-redoxin	−1.838
CS401_RS09340	AAA family ATPase	−1.812
CS401_RS06480	Ribonucleoside-diphosphate reductase subunit alpha	−1.802
CS401_RS03235	Mechanosensitive ion channel	−1.781
CS401_RS08655	3-keto-5-aminohexanoate cleavage protein	−1.735
CS401_RS01275	HAD-IB family hydrolase	−1.723
CS401_RS07720	Hypothetical protein	−1.719
CS401_RS02565	WGR domain-containing protein	−1.702
CS401_RS04775	DUF1871 family protein	−1.692
CS401_RS0675	Ribonucleotide-diphosphate reductase subunit beta	−1.677
CS401_RS07725	Hypothetical protein	−1.637
CS401_RS08570	4-methyl-5(B-hydroxyethyl)-thiazole monophosphate biosynthesis protein	−1.635
CS401_RS01055	DUF1456 family protein	−1.615
CS401_RS09170	thiol:disulfide interchange protein	−1.579
CS401_RS06365	Hypothetical protein	−1.573
CS401_RS05800	ATP-dependent DNA helicase RecG	−1.549
CS401_RS08910	Osmolarity sensor protein EnvZ	−1.547
CS401_RS00170	glutamate dehydrogenase	−1.535

*co/mono: coculture/monoculture.

**Figure 4.**
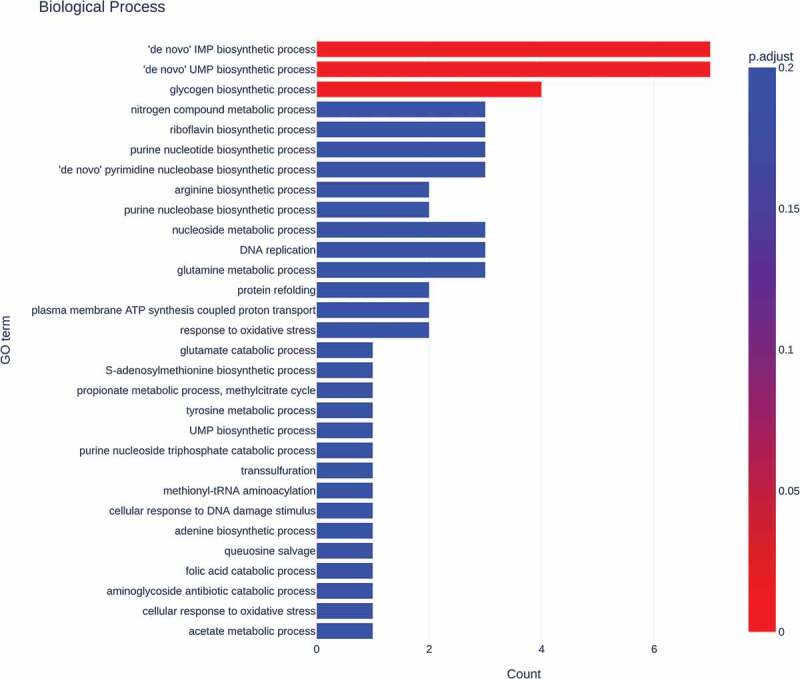
Gene ontology (GO) enrichment of differentially expressed genes. Gene count from GO in biological process. Gradient from red to blue on the bar indicates the adjusted p-value.

The GO enrichment analysis of the 139 differentially expressed genes showed significant changes in *de novo* synthesis of inosinic acid and uridylic acid and glycogen biosynthesis in biological process (*p* < 0.05, Figure 4).

## Discussion

In the present study, the effect of *P. gingivalis* diffusible signaling molecules on *F. nucleatum* was examined to clarify the synergy between these microorganisms involved in a dysbiotic shift of the dental plaque microbiome. *F. nucleatum* cocultured with *P. gingivalis* enhanced biofilm formation, and 139 differentially expressed genes were detected compared with those in monocultured *F. nucleatum*, including the ligand for adherence (*fap2)* to *P. gingivalis*. The GO analysis suggested sugar metabolism shift to glycogen biosynthesis, whereas the *de novo* purine and pyrimidine synthesis were decreased.

The expression of *fap2* increased significantly in cocultured *F. nucleatum*. Fap2 is a galactose inhibitable adherence factor involved in coaggregation between *F. nucleatum* and *P. gingivalis*, hemagglutination by *F. nucleatum* [17], and localization of *F. nucleatum* to colon tumors [36]. The results indicate that the sensing of diffusible signaling molecules from *P. gingivalis* by *F. nucleatum* induces an increase in *fap2*. The increase in Fap 2 may contribute to binding between the species and enhance these intimate interactions, although further analysis is required to confirm the magnitude of contribution. Fap2 is also involved in hemagglutination by *F. nucleatum* [17]. *P. gingivalis* has hemagglutination activity, which is involved in heme acquisition [37]. In the cocultured *F. nucleatum*, *cobK* and *cbiJ* expression increased. These genes are involved in porphyrin metabolism. It is possible that hemagglutination is involved in iron acquisition in *F. nucleatum*. Therefore, the increase in Fap2 expression may be one of the adaptations for establishing a synergistic community of *F. nucleatum* and *P. gingivalis.*

Increased expression of genes involved in the glycogen synthase pathway in cocultured *F. nucleatum* was detected through the GO analysis, including those encoding GlgG, glucose-1-phosphate adenylyltransferase, GlgB, and GauU. The increase in GlgG and glucose-1-phosphate adenylyltransferase expression in *F. nucleatum* biofilms formed during coculture with *P. gingivalis* compared with their expression in a single-species biofilm of *F. nucleatum* has also been reported in a proteomic study [38]. GalU also plays an important role in glycogenesis and cell wall synthesis. Mutant *galU* affects biofilm formation in *Escherichia coli* [39]. In *Pseudomonas aeruginosa*, *galU* is required for lipopolysaccharide-core organization [40]. In *Xanthomonas citri*, *galU* is involved in exopolysaccharide and capsular polysaccharide synthesis [41]. *galU* is also essential for biofilm formation in *Vibrio cholerae* [42]. In *F. nucleatum*, the major energy source is amino acid catabolism; however, this microorganism can also metabolize fructose [43]. The study showed that fructose metabolism is stopped, and polysaccharides are organized under amino-acid-rich conditions. *P. gingivalis* has Arg-gingipain; Lys-gingipain [44,45]; dipeptidyl peptidases such as DPP4, DPP5, DPP7, and DPP11; as well as serine exopeptidases [46]. The strong proteolytic activity of *P. gingivalis* is considered to enhance *F. nucleatum* growth [12]. Here, the expression of genes involved in amino acid metabolism was not altered, but Hendrickson et al. [38] reported that the coculture of *P. gingivalis* and *F. nucleatum* inhibited more numbers of amino acid metabolic pathways compared with *F. nucleatum* alone. *F. nucleatum* and *P. gingivalis* were incubated in PBS for 18 h in their study, whereas in this study, microorganisms were inoculated in TSBhm for 2 days, and therefore, we observed large amounts of amino acids. In addition, the subspecies of *F. nucleatum* used were different between the studies. These differences in the experimental conditions may contribute to the difference in the results. The increase in the expression of genes involved in glycogen synthesis strongly suggested that the shift of energy source from sugar to amino acid was promoted by the increase in the levels of available amino acids upon coculture with *P. gingivalis.*

In contrast, the expression of *pelF* and *pelG* in *F. nucleatum* was reduced in the coculture. *pelF* and *pelG* are involved in the synthesis and transport of the exopolysaccharides Psl and Pel, and their protein production is regulated *via* cyclic-di-GMP in *P. aeruginosa* [47,48]. It is possible that the downregulation of these genes results in an increase in biofilm formation in cocultured *F. nucleatum*, although further analysis of the role of these genes in *F. nucleatum* is required.

Fusolisin, which is an autotransporter protease [49], showed increased expression in cocultured *F. nucleatum*. The expression of branched-chain amino acid transporter permease, basic amino acid ABC transporter substrate-binding protein, and amino acid ABC transporter ATP-binding protein was also increased in cocultured *F. nucleatum*. As mentioned earlier, *P. gingivalis* shows a strong proteolytic activity. These genes may be involved in peptide digestion or amino acid import. However, in previous proteomic analyses, the expression levels of these transporters did not change in *F. nucleatum* cocultured with *P. gingivalis* [38,50] or *S. gordonii*. In the previous studies, *F. nucleatum* was cocultured without a membrane, whereas in this study, *F. nucleatum* was cocultured with *P. gingivalis* separated by an 0.4-µm pore membrane. One study used Brucella broth [50] and another used Todd Hewitt broth [38]. In addition, in the former study, the samples of biofilms were harvested after 4 days of culture, and in the latter, samples were obtained after 18 h of incubation in PBS; in this study, we harvested samples after 2 days. This might explain the differences in the results.

Genes for d*e novo* synthesis of inosinic acid and uridylic acid were downregulated in our study. These processes involve the synthesis of pyrimidine and purine using amino acids. Previous proteomic studies have found only minor changes in the proteome of *F. nucleatum* cocultured with *P. gingivalis*, primarily a decrease in the production of specific proteins [50]. In the present study, the numbers of decreased and increased proteins were comparable.

Here, the expression of genes involved in riboflavin synthesis from GTP was reduced, such as *ribD* and the riboflavin synthase gene. In eukaryotic cells, metabolic convergence of glutamine toward nucleotide biosynthesis observed in the process of malignant progression and inhibition of the shift reduced the proliferation of cancer cells [51]. It is possible that these reductions associated with purine and pyrimidine reflect changes in the external conditions caused by *P. gingivalis*, including the amino-acid-rich condition induced by the proteolytic activity, and the downregulation of genes involved in nucleotide biosynthesis from glutamine supports the glutamine for energy production. Furthermore, *P. gingivalis* and *F. nucleatum* produce indole that induces increased biofilm formation [52]. The biofilm mass in cocultured *F. nucleatum* was almost double that in the monoculture. In the biofilm, gradients of nutrients and oxygen induce a gradient of growth rate that results in fast-growing cells at the surface and slow-growing cells in the deeper layer of the biofilm [53]. It is possible that *F. nucleatum* growth decreases with increased biofilm formation and, therefore, the expression of the enzymes involved in pyrimidine and purine synthesis is decreased.

The expression of chaperones, including ClpB, DnaK, and DnaJ, was increased in the cocultured *F. nucleatum*. Although chaperones are generally cytoplasmic, six chaperones, including DnaK and ClpB, were detected in the matrix of *F. nucleatum* monoculture biofilm [54]. This indicates a relationship between chaperone translocation to the biofilm matrix and increased expression of these genes in *F. nucleatum* stimulated by *P. gingivalis* in coculture.

SDS-PAGE and immunoblotting indicated an increase in FomA expression in cocultured *F. nucleatum*. In the RNA-sequencing analysis, *fomA* expression did not show a difference between monoculture and coculture. FomA is a major porin protein in the matrix of biofilms formed by *F. nucleatum* [54]; however, an increase in FomA expression has not been observed in other coculture studies [55]. A recent study reported that the small RNA FoxI acts as a post-transcriptional repressor of FomA induced by oxygen [56]. Therefore, post-transcriptional regulation by FoxI or turnover of the protein in *F. nucleatum* cells may be responsible for the increase in FomA expression. In this analysis, an internal control was not evaluated, although the quantity of the protein was adjusted and the bands on SDS-PAGE showed almost identical levels. Therefore, further analysis using an internal control is required.

In the present study, *F. nucleatum* TDC 100 was used because of its strong synergy with *P. gingivalis*. The result of RNA-sequencing showed that this strain belongs to *F. nucleatum* subsp. *vincentii*. Previous studies on such gene or protein expression profiling used *F. nucleatum* subspecies *nucleatum*. Subspecies may show differing characteristics, including biofilm formation [57]. Human skeletons from the 18^th^ and 19^th^ centuries show high abundance of *F. nucleatum* subsp. *vincentii* with *P. gingivalis* and *Prevotella pleuritidis*, and this is maintained in modern samples [58]. *F. nucleatum* subsp. *vincentii* and *P. gingivalis* are useful for diagnosing periodontitis in mass patient screening using saliva samples [59]. High abundance of *F. nucleatum* subsp. *vincentii* is associated with periodontal lesions, and it further highlights the importance of this subspecies in the periodontopathic microbiome. To fully understand *F. nucleatum*-associated dysbiotic shift, further analysis of gene expression using multiple subspecies is required.

In this study, the gene expression was evaluated at a single time point before stationary phase, and therefore a single growth phase, of *F. nucleatum*. Several factors, other than coculture with *P. gingivalis*, such as differences in the growth phase, affect gene expression. To elucidate the influence of coculture, the evaluation of gene expression at multiple time points and in different ratios between the bacteria is required. TSBhm was used in the current assay; in other proteomics analyses, Brucella broth [50] or Todd-Hewitt broth [38] containing hemin and menadione were used. In the latter studies, the investigation was conducted in PBS. These media can be used to detect synergistic interactions; however, differences in the medium content may result in different results. In addition, a precise investigation on the synergistic effect requires the conditions that simulate those in the oral cavity. To fully comprehend the synergism between *F. nucleatum* and *P. gingivalis*, additional research using media that mimic the physiological condition of the oral cavity is required.

In conclusion, the diffusible signaling molecules from *P. gingivalis* induced an increase in the ligand for coaggregation with *P. gingivalis* and led to metabolic changes, including the promotion of polysaccharide synthesis and inhibition of the *de novo* synthesis of purine and pyrimidine in *F. nucleatum*. The metabolic changes may play a key role in the acceleration of biofilm formation by *F. nucleatum* cocultured with *P. gingivalis*. Therefore, the metabolic pathway in which differentially expressed genes involved in this study could be used as potential therapeutic targets for preventing dysbiosis.

## Data Availability

The sequence raw data obtained using RNA-Sequencing analysis has been deposited to the Sequence Read Archive in the DNA Data Bank of Japan (DDBJ/DRA) (https://www.ddbj.nig.ac.jp/dra) under submission ID: DRA014805.
